# A Method for the Design and Development of Medical or Health Care Information Websites to Optimize Search Engine Results Page Rankings on Google

**DOI:** 10.2196/jmir.2632

**Published:** 2013-08-27

**Authors:** Suzanne Dunne, Niamh Maria Cummins, Ailish Hannigan, Bill Shannon, Colum Dunne, Walter Cullen

**Affiliations:** ^1^Graduate Entry Medical SchoolUniversity of LimerickLimerickIreland; ^2^Centre for Interventions in Infection, Inflammation and Immunity (4i)Graduate Entry Medical SchoolUniversity of LimerickLimerickIreland

**Keywords:** health care information, patient education, Google, Internet, medical informatics, generic drugs, website development, quality assessment

## Abstract

**Background:**

The Internet is a widely used source of information for patients searching for medical/health care information. While many studies have assessed existing medical/health care information on the Internet, relatively few have examined methods for design and delivery of such websites, particularly those aimed at the general public.

**Objective:**

This study describes a method of evaluating material for new medical/health care websites, or for assessing those already in existence, which is correlated with higher rankings on Google's Search Engine Results Pages (SERPs).

**Methods:**

A website quality assessment (WQA) tool was developed using criteria related to the quality of the information to be contained in the website in addition to an assessment of the readability of the text. This was retrospectively applied to assess existing websites that provide information about generic medicines. The reproducibility of the WQA tool and its predictive validity were assessed in this study.

**Results:**

The WQA tool demonstrated very high reproducibility (intraclass correlation coefficient=0.95) between 2 independent users. A moderate to strong correlation was found between WQA scores and rankings on Google SERPs. Analogous correlations were seen between rankings and readability of websites as determined by Flesch Reading Ease and Flesch-Kincaid Grade Level scores.

**Conclusions:**

The use of the WQA tool developed in this study is recommended as part of the design phase of a medical or health care information provision website, along with assessment of readability of the material to be used. This may ensure that the website performs better on Google searches. The tool can also be used retrospectively to make improvements to existing websites, thus, potentially enabling better Google search result positions without incurring the costs associated with Search Engine Optimization (SEO) professionals or paid promotion.

## Introduction

A multitude of studies have assessed the use, quality, and availability of medical/health care information on the Internet in areas as diverse as inflammatory bowel disease [1], orthodontistry [2,3], pain [4], cancer [5-7], and mental health [8,9], among many others. Such studies often look at information available to, and used by, people in particular geographic areas, for example, pediatric asthma in Saudi Arabia [10], preconception care in Italy [11], and medical information in Brazil [12] and Portugal [13]. A PubMed search for research into online medical information, including, for example, use of resources such as Wikipedia or Google in medical education and availability of information for patients, provides thousands of search results. This is indicative of the fact that the Internet has become a source of medical information for patients and health care professionals alike, as shown by the increasing prevalence of the Internet use and social networking associated with “Web 2.0” for information sourcing and sharing online [14].

In the area of generic medicines, misconceptions and misinformation exist that are easily disseminated and perpetuated online. Given that health care professionals have expressed poor opinions of generics in the past [15], it is therefore challenging to communicate accurate information to the general public about the medicines that they are taking. There is a need to provide accurate information, to dispel myths, and to counter misinformation, but also to present the material in a manner that is accessible to the intended audience. For example, it has been reported that, in the case of patients particularly, myths and uncertainties about generic medicines abound and that accurate information can be difficult to come by [16].

A good quality medical or health care information website could be defined as one that contains accurate and unbiased information on all aspects of the topic (both positive and negative) for which the website is published, in conjunction with the ability of the website to be easily read and understood by its target audience. Where the audience is intended to be the general public, readability of the website will be a key factor in its success (as defined by the number of hits the website receives, indicative of its ranking on Internet search engine results). After all, if a website contains exemplary information but cannot be easily read and understood by its audience, it is possible for it to go largely undiscovered in the plethora of information available on the Internet. This study focused on non-advertised or promoted websites (ie, rankings on a Search Engine Results Page (SERP) that are not there as a result of a paid advertisement or promotion but rather are ranked and returned by Google’s algorithms).

While the availability and accuracy of existing online medical/health care information continues to be studied, much less work appears to have been performed in the area of development of medical information websites—in particular websites aimed at providing accurate and unbiased medical information to the general public. A PubMed search done February 22, 2013, using the search term *development medical information website,* returned 28 articles specifically related to the topic of development of medical/health care information websites.

The objective of this paper was to provide a method for the planning of information to be included in medical information websites and for representing that information in a readable manner. As Search Engine Optimization (SEO) can be a critical factor in ensuring top-ranking search engine results [17] and given that the cost of using potentially expensive online advertising or SEO professionals in order to promote a website may be prohibitive for government or advocacy groups wishing to impart good quality medical/health care information, use of the tools and techniques described in this paper will not only ensure the quality of the information in the website but may also provide the website with an improved chance of being returned to a searcher in a higher ranking on a Google SERP, without incurring significant additional cost.

## Methods

### Rationale

To ensure a high-quality medical information website, two factors should be considered in its development: (1) the information it will present (quality, accuracy, comprehensiveness, balance, impartiality, etc) and (2) the ability of the information to be read and understood by the target audience.

Based on these factors, an assessment tool was developed that may be used to prospectively design the content of an optimized website. This study reports the composition of that tool and its validation through retrospective assessment of existing sites.

### Information Gathering and Website Quality Assessment Tool Development

A tool for assessment of websites imparting information on generic drugs was developed. This Website Quality Assessment (WQA) tool consisted of a series of yes/no type questions, where a point was awarded for positive or correct information (see Table 1). No points were awarded for information lacking or for inaccurate information. Questions that cannot be answered were designated “not applicable” (N/A) and no score awarded. An overall WQA score for each website was totaled from the scores assigned to each assessment question.

In the development of the WQA tool, the following criteria were used:

1. Is there a listing of the questions likely to be asked by the searcher?
2. What myths or misinformation exist on the topic that may need to be dispelled or corrected?
3. What information could be required by the searcher in order to assist in making informed decisions?
4. Are there relevant comparisons or analogies that might help in understanding of the topic by a nonscientist or clinician?
5. Is there any associated or corollary information from other related topics or areas that might be helpful to support understanding of the topic?

The number of assessment questions will be determined by the topic in question and is not fixed. However, all areas in the 5 criteria steps noted above should be covered in the WQA questions used.

**Table 1:** Website Quality Assessment for assessing information on websites on generic medicines.

Question	Answer and score
Does the site explain what a generic medicine is?	Yes=1 No=0
Is this explanation correct?(ie, equivalent in dose, strength, route of administration, safety, efficacy, and intended use)	Yes=1 No=0
If so, is the explanation of a generic medicine readable and understandable by a nonscientist?	Yes=1 No=0
Are examples given of generic medicines? Eg, example of a proprietary medicine that also states the counterpart generic medicine?	Yes=1 No=0
Is bioequivalence mentioned in the website?	Yes=1 No=0
Is bioequivalence explained?	Yes=1 No=0 N/A
If so, is the explanation of bioequivalence correct?	Yes=1 No=0 N/A
If so, is the explanation of bioequivalence readable and understandable by a nonscientist?	Yes=1 No=0 N/A
Is the cheaper price of generics mentioned?	Yes=1 No=0
Is an accurate reason for the cheaper price of generics given?	Yes=1 No=0 N/A
Is any inaccurate information regarding the cheaper price of generics given?	Yes=0 No=1 N/A
Are examples given of the actual price difference between generics and proprietary medicines, or of the amount of money that can be saved by use of generics?	Yes=1 No=0
Is reference made to the fact that approved, equivalent generic meds can have a different appearance (color, shape, etc) different taste/smell or different inactive ingredients?	Yes=1 No=0
Are narrow therapeutic index (NTI) drugs mentioned?	Yes=1 No=0
Is the difference between NTI and non-NTI drugs explained?	Yes=1 No=0 N/A
Is there accurate information given on how generic bioequivalence or generic manufacturing may affect NTI drugs?	Yes=1 No=0 N/A
Is any inaccurate information given regarding NTI drugs?	Yes=0 No=1 N/A
Are “pros” of generics mentioned? (eg, lower price for same safety and bioequivalence, etc)	Yes=1 No=0
Are any “cons” of generics mentioned? (eg, adverse events to dissimilar excipients, etc)	Yes=1 No=0
Is the difference between proprietary and nonproprietary names mentioned?	Yes=1 No=0
Is the explanation given for the difference between proprietary and nonproprietary names accurate?	Yes=1 No=0 N/A
Is generic prescribing mentioned and explained accurately?	Yes=1 No=0
Total WQA score	
Flesch Reading Ease score	
Flesch-Kincaid Grade Level	

### Validation of the WQA Tool

To validate the tool, all searches were performed on Google (google.com) and a number of the resulting hits in the SERPs returned were assessed using the 22-question Generic Medicines WQA (Table 1). The search was physically done in several English-speaking countries, using computers with Internet protocol (IP) addresses in those countries, in order to determine if there was any country-to-country (or geographic) variability. The searches were performed in the United States, Canada, Ireland, Great Britain, and Australia. The search term used was identical in all cases: “generic drug OR medicine” (without the quotes). All searches were performed during March and April of 2012, and a total of 24 distinct websites were assessed.

To measure reproducibility of use of the tool, each of the websites was independently assessed by 2 different raters.

### Assessment of Website Readability

Readability of text is an important issue, especially in the medical domain. For this study readability of text was assessed using two methods: (1) Flesch Reading Ease score and (2) Flesch-Kincaid Grade Level. However, it is worth noting that other readability evaluation methods have also been used in the assessment of medical texts [18].

A minimum of a 100-word sample of continuous text was selected at random from the website text and pasted into Microsoft Word. This text was then analyzed using the readability statistics in the MS Word application.

MS Word’s Flesch Reading Ease score is based on a formula developed in 1948 by Rudolf Flesch [19]. It is computed using the average number of syllables per word and words per sentence. Syllables-per-word is a measure of word difficulty. Words-per-sentence is an indicator of syntactic complexity.

The Flesch Reading Ease scale ranges from zero to 100. Zero to 50 is very difficult to difficult reading. Eighty and above is easy to very easy reading. Flesch himself set the minimum score for plain English at 60 [19]. Microsoft’s documentation encourages authors of standard documents to aim for a score of 60 to 70 [20,21].

The Flesch-Kincaid Grade Level, which was developed in 1975, measures the readability of a document based on the minimum education level required for a reader to understand it [22]. Microsoft recommends aiming for a Flesch-Kincaid score of 7.0 to 8.0 for most documents. According to a 1993 study, the average adult in the United States reads at the seventh-grade level and the authors of that study recommended that materials for the public be written at a fifth- or sixth-grade reading level [20].

### Statistical Analyses

The mean and standard deviation of the differences between the 2 reviewers for all three tools (WQA, Flesch Reading Ease score, and Flesch Kincaid Grade Level) were used to calculate limits of agreement, which are represented graphically in Bland-Altman plots. The intraclass correlation coefficient (ICC) was used to measure reproducibility. Spearman correlation coefficient (*r*_s_) was used to measure the association between the ranking of websites with WQA scores and readability assessments. Absolute values of *r*_s_>0.3 were considered to represent moderate correlations; >0.5 were considered strong correlations. The scores from the developer of the assessment tool (SD) were used in the correlation analyses. The correlation between ranking of websites and WQA scores was also used to demonstrate the predictive validity of this newly developed assessment tool.

## Results

### Validation of the WQA Tool

Statistical analysis of the 2 independent raters (SSD and NC) using Bland-Altman plots showed that, for WQA assessments of the websites, the mean difference (SSD minus NC) represented by the solid black line in a) in Multimedia Appendix 1was zero (SD 1.18) indicating perfect agreement on average. The median difference was also zero (range –3 to 2). Only one observation was outside the limits of agreement (this website was a list of brand name medicines alongside the names of their generic counterparts). One rater performed the WQA based on this list, whereas the second rater looked for information on other pages of the website, thus accounting for the difference in WQA ratings awarded. An ICC value of 0.94 indicated excellent reproducibility between different users.

Similar analysis of the readability of the websites using Flesch Reading Ease score (on a scale of 0 to 100) and Flesch-Kinkaid Grade Level (on a scale of 1 to 18) showed comparable levels of agreement (see b) and c) in Multimedia Appendix 1. The mean difference (SSD minus NC) for reading ease score is 4.66 (SD 12.06) indicating that rater SSD was scoring slightly higher than NC on average. The mean difference (rater SSD minus NC) for grade level was -1.79 (SD 2.86) indicating that rater SSD was scoring slightly lower than NC on average. One observation in each case was outside the limits of agreement. However, as each rating was independent, different sections of text were likely to be taken from each of the websites assessed. This variation in the text taken most likely accounted for the single observation outside the limits of agreement. An ICC value of 0.71 for Flesch Reading Ease score and 0.63 for Flesch-Kincaid Grade Level demonstrate moderate to strong reproducibility, particularly given the subjectivity of this type of assessment, and the possible variability in the text selected by reviewers for assessment.

Overall, the WQA and readability scores demonstrate acceptable reproducibility of the tools when by used by more than 1 rater.

### Correlation Between WQA Score and SERP Ranking

Scatterplots of WQA score against rankings on Google SERPs in different regions worldwide (United States, Canada, Ireland, United Kingdom, and Australia) are given in Multimedia Appendix 2. Using Spearman correlation coefficient, a moderate to strong correlation between a WQA score and ranking on Google SERPs could be seen (Table 2). The observed relationship was seen in Google searches done in the different regions worldwide indicating that the correlation occurs regardless of the location or IP address of the searcher’s computer. The strongest correlation (*r*_s_=-0.67), was seen in the Google search performed in the United States.

Therefore, use of WQA assessment questions while developing information for inclusion in a medical information website could, by corollary, be a step towards ensuring higher Google SERP rankings and, therefore, exposure to a greater potential audience for the website.

### Correlation of Readability With SERP Ranking

There was also a relationship, in general, between readability and ranking on Google searches (Table 2). Flesch Reading Ease scores were correlated with the SERP ranking of the websites in each country. Again, the strongest relationship was seen in the US Google search (*r*_s_=-0.64). In general, the top ranked sites (placed 1, 2, etc) tended to have the higher Reading Ease scores. Because of the small sample sizes in the study (at most 10 websites in each domain) and hence low statistical power, a descriptive analysis is presented and no hypothesis tests were carried out.

Additionally, scores for Flesch-Kincaid Grade Level assessments were correlated with SERP ranking of the websites. In general, the top ranked sites tended to have lower grade level values with the most significant relationship again being seen in the US search (*r*_s_value of 0.68). Therefore, the implication is that that websites with greater ease of readability are more likely to rank high in, and therefore be accessed from, Google SERPs.

**Table 2:** Correlation between WQA, reading ease score, and grade level with ranking using Spearman correlation coefficient (*r*_s_).

Domain	n	WQA, Spearman *r*_s_	Flesch Reading Ease score, Spearman *r*_s_	Flesch-Kincaid Grade Level, Spearman *r*_s_
US / .com	7	-0.67	-0.64	0.68
CA / .com	8	-0.38	-0.48	0.43
IE / .com	8	-0.49	-0.33	0.24
UK / .com	8	-0.38	-0.48	0.43
AU / .com	8	-0.34	0.29	-0.38

## Discussion

### Principal Findings

Prior to publication of a website, information must be gathered and written that will be disseminated to the intended audience through the website. Development and use of a specific WQA-type assessment during the design phase of a medical/health care information website on any topic will ensure that the information put into the website is of sufficient quality to satisfy potential searchers and users of the website. WQA can be used to assess drafts of the information to be published. Use of positive and negative scoring (positive scoring for information that is necessary, of good quality, and needed to support the integrity of the website; negative scoring for any information that is inaccurate, biased, or that may take from the integrity of the information) employed by WQA assessment ensures that all aspects of the information gathering initiative are accounted for during the website design.

As the Internet is one of the first places a patient is likely to go when searching for medical information [23] and given that Google is the primary search engine in use worldwide, holding almost 90% of the global search engine market [24], corollary use of WQA could possibly lead to higher rankings on Google SERPs for websites using this tool in their design and development.

Furthermore, this study has demonstrated that websites with greater ease of readability are more likely to rank high in, and therefore be accessed from, Google searches. Therefore, inclusion of Flesch Reading Ease and Flesch-Kincaid Grade Level assessments as part of the WQA enable a more comprehensive assessment of how the website might perform in Google searches. We have demonstrated in this paper that high readability scores and WQA scores are more likely to lead to a high Google SERP ranking.

### Limitations

A limitation of this study is the small number of websites assessed. Further studies in this area could make use of technology, for example, a web crawler to gain additional information that could allow for clustering or commonalities across a spectrum of similar websites to be examined. A further study could evaluate sites containing similar content but focus instead on usability and accessibility, for example, are the sites well designed, are they pleasing to the eye, and is the navigation user-friendly? Isolating such content from the design and visual presentation of websites would provide further insight into the usability and accessibility of medical information providing websites that would complement the findings in this paper. Indeed, information from such a study, if done using websites focused on generic medicines, may provide insight into the adoption and penetration of such medicines in different markets worldwide.

Readability formulas, additionally, have limitations in that a favorable score may not always be fully indicative of clarity of information (for instance, not all low-syllable words are always clearly understood, shorter sentences are not always necessarily easier to read, and inferences may be required that may increase the complexity of the text). Therefore, these formulas need to be used in conjunction with other plain language guidelines when writing for provision of health care information (especially for low literacy and limited English proficiency audiences), and not used as sole measures of understandability.

### Conclusions

With about 16% of adults in the United Kingdom being described as “functionally literate” (ie, they have literacy levels at or below those expected from an 11-year old [25]), and the International Adult Literacy Survey showing that 1 in 4 adults in the Republic of Ireland have problems with even the simplest of literacy tasks [26] (with similar rates being seen in the United States [27] and Canada [28]), it is fair to say that writing of medical information websites with this in mind may be the most important aspect in providing medical information to the general public. This point, of course, applies to all printed material (eg, pamphlets given to patients), not just information published online. Arguably, it follows that training writers of medical information (to be disseminated to the general public, for instance) in methods of presenting simple, clear language is an important aspect in ensuring that the general public understand the information that health care professionals might be trying to impart to them. This becomes particularly important in light of research showing that there is often a discrepancy between the information that a physician believes a patient to have and what the patient actually understands [29].

Language complexity as a block to accessibility of information has been recognized by Wikipedia, the 6^th^most commonly accessed website in the world [30] and, as a solution, Wikipedia is available in both English and Simple English, where the Simple version is intended to be more accessible by use of simplified language and limited vocabulary. Consequently, Wikipedia guidelines on writing of the Simple version may be of use to those creating medical information websites for the general public [31].

Overall, use of the WQA tool in the planning and preparation of material for medical information websites, alongside an assessment of readability of the written material, is likely to ensure that the website subsequently ranks higher in Google SERPs and is thus more likely to be accessed, as well as read and understood, by the intended audience.

## Multimedia Appendix 1

Bland-Altman plots for WQA, Flesch Reading Ease Score, and Flesch Kincaid grade level.

## Multimedia Appendix 2

Scatterplots of WQA score against rankings on .com domains.

## Abbreviations

ICC: intraclass correlation coefficient
IP: Internet protocol
SEO: search engine optimization
SERP: search engine results page
WQA: website quality assessment
