# Supplementary material for: A Method for the Design and Development of Medical or Health Care Information Websites to Optimize Search Engine Results Page Rankings on Google
Source: J Med Internet Res. 2013 Aug 27;15(8):e183. doi: 10.2196/jmir.2632 (PMC3758043; doi:10.2196/jmir.2632)

Multimedia Appendix 1. Bland-Altman plots for WQA, Flesch Reading Ease Score, and Flesch Kincaid grade level.

Mean difference between the 2 raters is represented by the solid black line, and the 95% limits of agreement (dashed lines) are calculated using mean difference ±2 (SD of the differences).

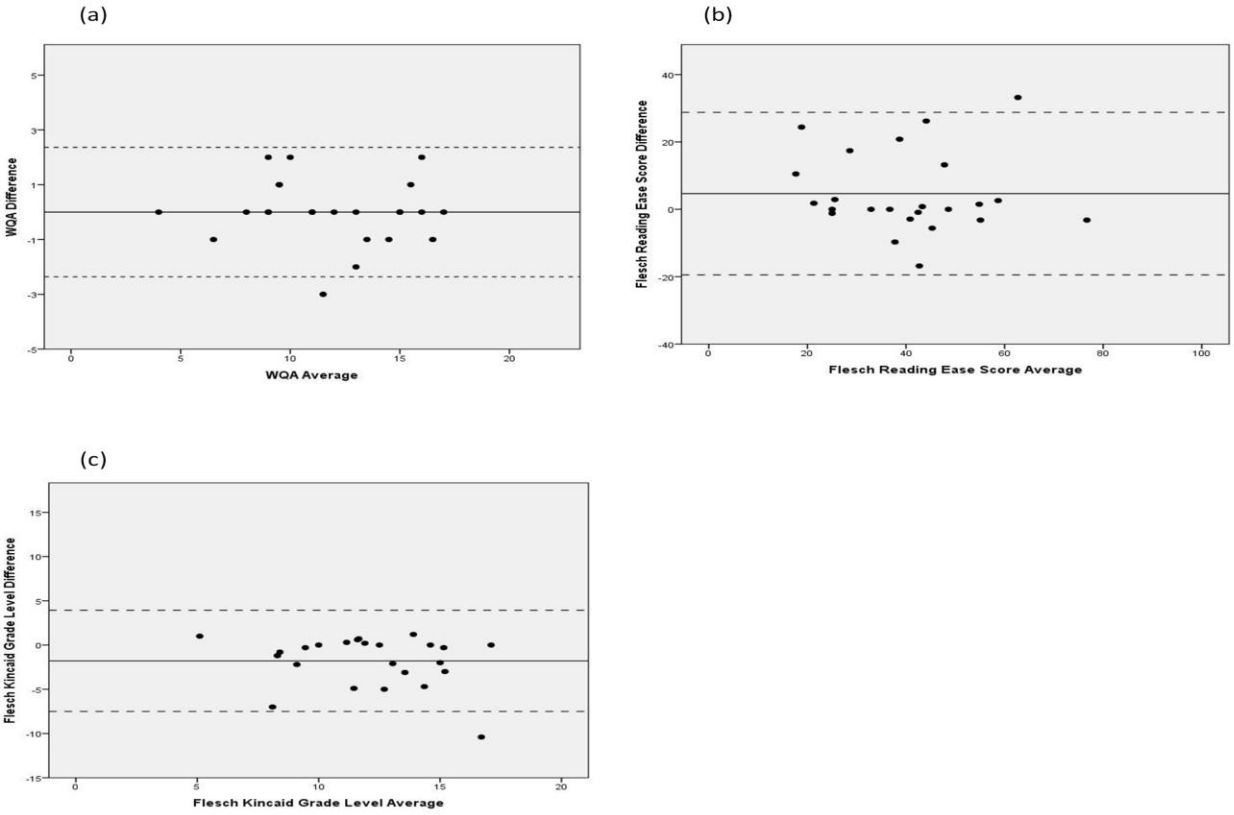

Supplement: Supplementary file 1 [file jmir_v15i8e183_app1.pdf]
