# Supplementary figures and images for: A Method for the Design and Development of Medical or Health Care Information Websites to Optimize Search Engine Results Page Rankings on Google
Source: J Med Internet Res. 2013 Aug 27;15(8):e183. doi: 10.2196/jmir.2632 (PMC3758043; doi:10.2196/jmir.2632)

**Multimedia Appendix 2.** Scatterplots of WQA score against rankings on .com domains.

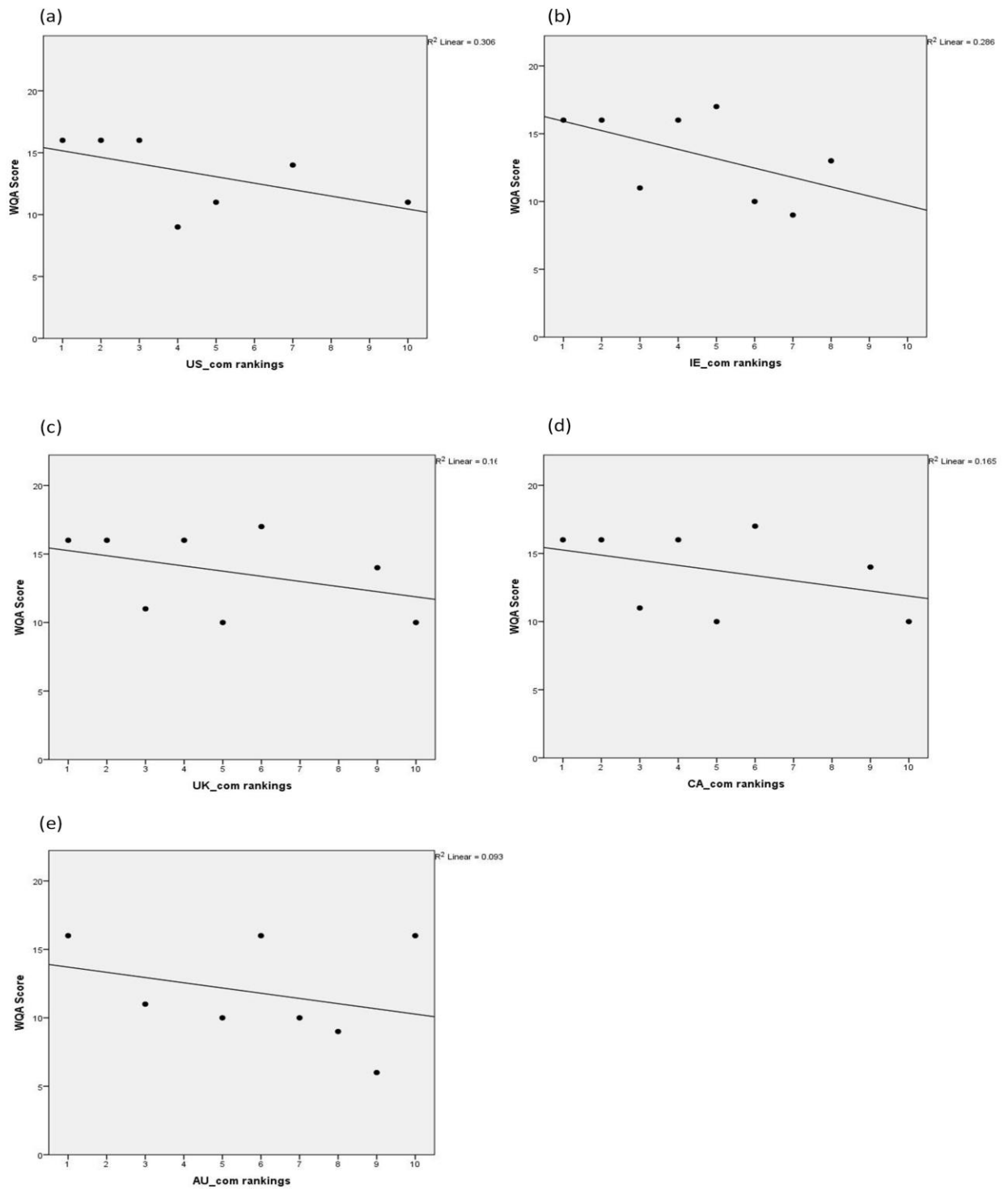

Supplement: Supplementary file 2 [file jmir_v15i8e183_app2.pdf]
